# Effects of a ketogenic diet on strength and power

**DOI:** 10.1186/1550-2783-11-S1-P41

**Published:** 2014-12-01

**Authors:** Sean A McCleary, Matthew H Sharp, Ryan P Lowery, Jeremy E Silva, Jacob T Rauch, Jacob A Ormes, Kevin A Shields, John I Georges, Jacob M Wilson

**Affiliations:** 1 The University of Tampa, Tampa, Florida, USA

## Background

The effects of a LCKD on endurance performance has been investigated several times, and it has already received a review. For this type of activity, no decrements in performance are observed once the participants are adjusted to the diet. However, LCKD’s are yet to be investigated in a resistance training model. One study has examined strength in relation to a LCKD, finding no decrements, yet this study failed to incorporate a well-controlled training protocol.

## Methods

The subjects all participated in the LCKD then all participated in the control diet 3 months later. They were reported as having 30 hours of training per week, although the training is not adequately described. All participants volunteered to participate in this study. Additionally over the course of three months, a significant training adaptation could occur. Thus, the results of this study are inconclusive. Additionally, data is lacking for LCKD’s in a healthy population. We aim to investigate the effects of a LCKD compared to a high carbohydrate diet, more typically used by athletes, on measures of athletic performance and perceived effects of exercise to eight weeks of periodized resistance training. Consent to publish the results was obtained from all participants.

## Results

For all strength and power measures there was a time effect. The 1RM bench press increased in both the LCKD(10.3±4.4kg) and western(9.5±4.0kg). The 1RM squat increased in both LCKD(12.7±5.9kg) and western(15.2±7.6kg). The wingate peak power increased in both the LCKD(51.8±64.7W) and western(80.5±66.8W).

## Conclusion

Both the LCKD and western group experienced an increase in bench press 1RM strength, squat 1RM strength, and wingate peak power. In the literature there is a lack of studies testing a LCKD diet on strength and power performance. For purely aerobic performance, there is no difference between high carbohydrate and high fat as long as a 3-4 week period for adaptation to a high fat diet is permitted . However, one study attempted to simulate a race-like environment, which incorporated anaerobic sprints during the aerobic event. This study found that no differences were present during the aerobic portion, but the time to travel 4km in the sprint was significantly greater in the ketogenic group. In our study we were able to demonstrate that a LCKD can produce similar strength and power gains to a western diet.

